# Clinical and imaging markers for the prognosis of acute ischemic stroke

**DOI:** 10.3389/fneur.2024.1345914

**Published:** 2024-02-29

**Authors:** Chenyang Pei, Che He, Han Li, Xiangying Li, Weihui Huang, Jun Liu, Jianzhong Yin

**Affiliations:** ^1^Tianjin Medical University, Tianjin, China; ^2^Department of Radiology, Haikou People's Hospital, Affiliated Haikou Hospital of Xiangya Medical College, Central South University, Haikou, Hainan, China; ^3^Medical Imaging Center, The First People's Hospital of Qujing, Qujing, Yunnan, China; ^4^Department of Neurology, Tianjin First Central Hospital, Tianjin, China; ^5^Department of Radiology, Tianjin Fourth Central Hospital, Tianjin, China

**Keywords:** acute ischemic stroke, NIHSS score, mRS score, diffusion, kurtosis, perfusion

## Abstract

**Background and purpose:**

Significant differences in the outcomes observed in patients with acute ischemic stroke (AIS) have led to research investigations for identifying the predictors. In this retrospective study, we aimed to investigate the relationship of different clinical and imaging factors with the prognosis of AIS.

**Materials and methods:**

All clinical and imaging metrics were compared between the good and poor prognosis groups according to the modified Rankin Scale (mRS) score at 90 days after discharge. Clinical factors included gender, age, NIHSS scores at admission, and other medical history risk factors. Imaging markers included the lesion’s size and location, diffusion, and perfusion metrics of infarction core and peripheral regions, and the state of collateral circulation. Spearman’s correlations were analyzed for age and imaging markers between the different groups. The Chi-square test and Cramer’s V coefficient analysis were performed for gender, collateral circulation status, NIHSS score, and other stroke risk factors.

**Results:**

A total of 89 patients with AIS were divided into the good (mRS score ≤ 2) and poor prognosis groups (mRS score ≥ 3). There were differences in NIHSS score at the admission; relative MK (rMK), relative MD (rMD), relative CBF (rCBF) of the infarction core; relative mean transit time (rMTT), relative time to peak (rTTP), and relative CBF (rCBF) of peripheral regions; and collateral circulation status between the two groups (*p* < 0.05). Among them, the rMK of infarction lesions had the strongest correlation with the mRS score at 90 days after discharge (r = 0.545, *p* < 0.001).

**Conclusion:**

Perfusion and diffusion metrics could reflect the microstructure and blood flow characteristics of the lesion, which were the key factors for the salvage ability and prognosis of the infarction tissue. The characteristics of the infarction core and peripheral regions have different effects on the outcomes. Diffusion of infarction core has strong relations with the prognosis, whereas the time metrics (MTT, TTP) were more important for peripheral regions. MK had a more significant association with prognosis than MD. These factors were the primary markers influencing the prognosis of cerebral infarction patients.

## Introduction

Acute ischemic stroke (AIS) is one of the common causes of death and disability worldwide (1). The recurrence rate is also high, which greatly reduces the patient’s quality of life. Many stroke survivors continue to experience dysfunction or long-term negative effects despite continuous rehabilitation (2). Therefore, stroke is a significant health problem and a financial burden on society (3). Prognostic prediction of AIS is essential to help patients and caregivers set appropriate clinical plans and monitor treatment during recovery and adaptation. In recent years, the mRS score has been used to evaluate neurological functional status after stroke (4), which can reflect characteristics of stroke recovery that are directly relevant to patients’ daily activities (5). Yet, it is challenging to predict neural function after an ischemic stroke. There are some potential indicators for long-term outcomes, such as age (6) and NIHSS score (4).

Magnetic resonance imaging (MRI) is commonly used for the diagnosis and evaluation of ischemic stroke, in which diffusion-weighted imaging (DWI) is particularly sensitive to reveal the range of cytotoxic edema (7, 8). Diffusion kurtosis imaging (DKI) complements the directionality of molecular diffusion (9). Both DWI and DKI could reveal the microstructure characteristics of the brain tissue (10). The lesion size obtained by DWI is an independent risk factor for the clinical outcome (11). Previous studies have shown that MD and MK closely relate to the necrotic regions at follow-up (12, 13). In addition, PWI provides the hemodynamic status of the infarct lesion and surrounding tissues. It can detect the severity and extent of the impaired perfusion for both the ischemic core and the surrounding brain regions (14, 15). Previous research has demonstrated a strong correlation between impaired brain perfusion and stroke recurrence and functional outcomes (16). MRA results can be used to evaluate the feeding artery of the lesion and can reflect the status of collateral circulation. Studies have found that the status of collateral circulation at the distal end of the lesion is significantly correlated with different prognoses (17).

This study aimed to investigate the correlation between the characteristics of an infarction, including the clinical and imaging features with its neurological outcome at the follow-up to explore the key factors in predicting the prognosis of an ischemic stroke.

## Methods

### Patients and groups

AIS patients admitted to the Tianjin First Central Hospital were recruited for the study. All patients had an apparent onset of neurological impairment (communication dysfunction, unilateral face or limb numbness, etc.). Acute cerebral infarction was confirmed by the admission MRI scan, which included T1WI, T2WI, DWI, DKI, Magnetic Resonance Angiography (MRA), and perfusion-weighted imaging (PWI). The clinical history, including demographics, risk factors, neurological evaluation, and NIHSS score (18), was collected. The NIHSS score ranged from 0 to 42, where 0–1 was defined as normal or nearly normal; 2–4 was considered mild; 5–15 was considered moderate; 16–20 was considered moderate–severe and 21–42 was defined as a severe stroke (19). The exclusion criteria were small lesions (minimum diameter < 10 mm), bilateral lesions, intracranial hemorrhage or lesions, craniocerebral trauma or surgery history, and endovascular or intravenous thrombolytic therapy.

**Figure 1:**
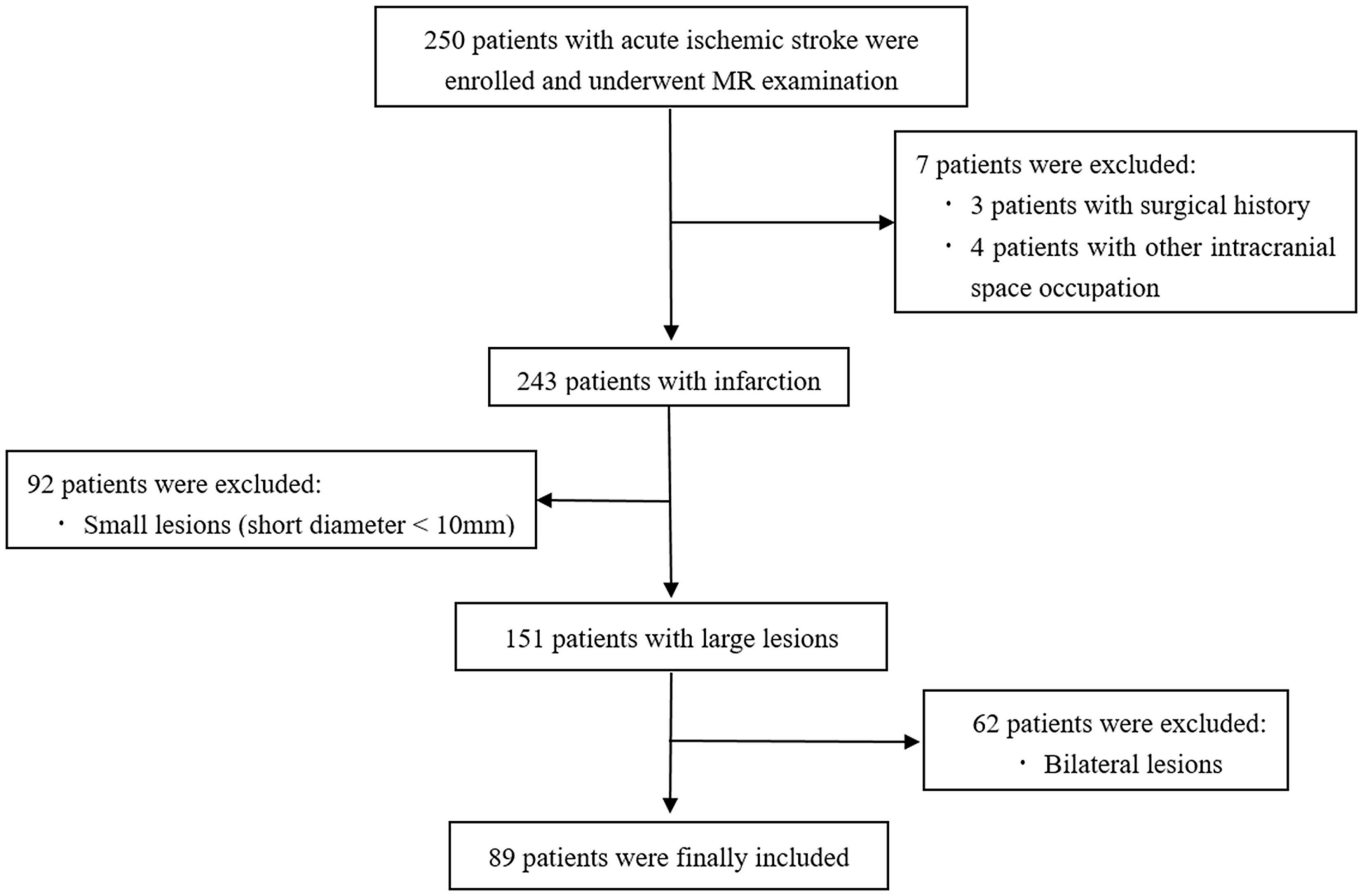
Flow chart of patients’ enrollment and exclusion processes.

During the study period, 250 AIS patients were collected. Of them, 161 patients were excluded due to other intracranial space occupation or history of craniocerebral surgery, bilateral cerebral infarction (62 patients), and the maximum diameter of DWI high signal <10 mm. Finally, 89 patients were enrolled in the present study (Figure 1). After 3 months of admission, the mRS score was assessed and used as an indicator of the prognosis of AIS (20), with mRS ranging from 0 (asymptomatic) to 6 (death). For the patients with mRS Score between 0 and 2, it indicated that the patients could live independently, while the patients with mRS Score higher than 2 would need different degrees of help from others (21–23). Therefore, 89 patients were divided into two groups: the good prognosis group and the poor prognosis group. The good prognosis was defined as mRS score ≤ 2, and the poor prognosis was defined as mRS score ≥ 3 at 90 days after discharge. Their demographic characteristics are shown in Table 1.

**Table 1:** The demographic, clinical and imaging features in different groups of patients (mean ± SD).

	All patients (*n* = 89)	Patients with good prognosis (n_1_ = 42)	Patients with poor prognosis (n_2_ = 47)	r	*P*
**Clinical risk factors**					
Age, y	61.854 ± 9.715	61.119 ± 9.793	62.511 ± 9.702	0.049	0.647
Gender	68/21	28/14	40/7	−0.217^#^	0.041^*^
NIHSS score	4.876 ± 3.867	3.357 ± 2.783	6.234 ± 4.208	0.372	<0.001^***^
Hypertension	72/17	35/7	37/10	0.059^#^	0.581
Diabetes	35/54	17/26	18/29	0.022^#^	0.834
Smoking	52/37	23/19	29/18	−0.07^#^	0.507
Drinking	24/75	9/33	15/32	−0.118^#^	0.266
Previous stroke history	21/78	12/30	9/38	0.111^#^	0.296
**Imaging metrics of infarction lesions**					
Lesion size (mm^3^)	3080.855 ± 5321.363	2179.490 ± 3897.603	3886.330 ± 6263.121	0.176	0.100
Types Cortex/Subcortical/Basal ganglia/Subtentorial	23/19/31/16	10/6/14/12	13/13/17/4	0.243^#^	0.188
rMD	0.621 ± 0.123	0.681 ± 0.117	0.566 ± 0.103	−0.449	<0.001^***^
rMK	1.496 ± 0.247	1.364 ± 0.173	1.614 ± 0.246	0.545	<0.001^***^
rMTT	1.480 ± 0.301	1.403 ± 0.273	1.549 ± 0.310	0.261	0.010^*^
rTTP	1.289 ± 0.170	1.232 ± 0.155	1.340 ± 0.169	0.325	0.001^**^
rCBF	0.552 ± 0.210	0.630 ± 0.213	0.482 ± 0.182	−0.348	<0.001^***^
rCBV	0.681 ± 0.214	0.739 ± 0.188	0.628 ± 0.224	−0.268	0.010^*^
Collateral circulation 0/1/2	38/33/18	13/12/17	25/21/1	0.477	<0.001^***^
**Imaging metrics of peripheral regions (*n* = 82; n**_**1**_ **= 38; n**_**2**_ **= 44, 7 brain stem infarctions had no peripheral regions)**					
rMD	1.002 ± 0.082	1.012 ± 0.113	0.994 ± 0.041	−0.071	0.525
rMK	1.003 ± 0.114	0.987 ± 0.156	1.017 ± 0.057	0.065	0.561
rMTT	1.117 ± 0.226	1.052 ± 0.169	1.174 ± 0.254	0.329	0.003^**^
rTTP	1.060 ± 0.117	1.026 ± 0.116	1.090 ± 0.111	0.332	0.002^**^
rCBF	1.037 ± 1.009	1.222 ± 1.460	0.876 ± 0.161	−0.278	0.011^*^
rCBV	0.937 ± 0.203	0.978 ± 0.139	0.902 ± 0.241	−0.125	0.263

^*^P < 0.05; ^**^P < 0.01; ^***^P < 0.001. ^#^Represents independent chi-square test and the correlation coefficient r obtained by Cramer’s V coefficient. For the remaining factors, Spearman correlation test was employed to get the r and *p*-values.

### MR protocol

All MRI examinations (including T1WI, T2WI, DWI, DKI, MRA, and PWI) were performed using a 3 T Siemens Trio imager with a 32-channel head coil (Siemens Healthineers, Erlangen, Germany). The DWI was performed using a single-shot echo-planar sequence with repetition time (TR)/echo time (TE) = 5,000/93 ms, field of view (FOV) = 221 × 211 mm^2^, matrix = 183 × 384, axial slices (number/thickness/gap) = 20/5.0 mm/1.25 mm, and three diffusion directions with two b values (0, 1,000 s/mm)^2^; DKI was performed using a multi-band echo-planar sequence with TR/TE = 3,000/95 ms, FOV = 230 × 230 mm^2^, matrix = 183 × 384, axial slices (number/thickness/gap) = 20/5.0 mm/1.25 mm, and 20 diffusion directions with three b values (0, 1,000, 2,000 s/mm)^2^. PWI was collected after the injection of intravenous contrast agent, TR/TE = 1,480/32 ms, FOV = 230 × 230 mm^2^, axial slices (number/thickness/gap) = 20/5 mm/1.25 mm; MRA sequence was performed using TOF-3D technology, TR/TE = 27/3.59 ms, FOV = 230 × 230 mm^2^, slice thickness = 0.7 mm; total scan time for the imaging protocol was about 15 min.

### Data processing

The DKI data were converted to the NIfTI format and then processed for motion correction, eddy current correction, and Gaussian smoothing noise reduction (FMRIB Software Library, http://www.fmrib.ox.ac.uk/fsl/). The mean diffusivity (MD) and mean kurtosis (MK) maps were derived from diffusional kurtosis estimator software.^1^

The PWI data were post-processed by Siemens “Neuro Perfusion” software package to obtain the mean transit time (MTT) (s) and the time to peak (TTP) (s), cerebral blood flow (CBF) (ml/min/100 g), and cerebral blood volume (CBV) (ml/100 g).

### Infarction lesions, peripheral regions, and collaterals evaluation

The volume of the lesions was obtained by the area of all slices multiplied by the slice thickness. All other imaging metrics of infarction lesions and peripheral regions were measured at the maximum slice of the lesions.

The lesions were delineated in MRIcron software, and the ROI of the maximum slice was saved and copied to the DKI and PWI maps to obtain MD, MK, MTT, TTP, CBF, and CBV values. In addition to the acute infarct lesions, the ROI were also flipped to the opposite side to obtain the above metrics of the contra-lateral normal brain regions. The relative metrics of diffusion and perfusion were the ratio between the lesion’s values and the contra-lateral normal values, referred to as the rMTT, rTTP, rCBF, rCBV, rMD, and rMK of the infarction lesions.

Besides the characteristics of the infarction lesions, the relative metrics (rMTT, rTTP, rCBF, rCBV, rMD, and rMK) of the peripheral regions outside the infarction were also measured. The ROI of the peripheral region would be within the 2 cm outside the lesions and at the same blood supply region of the infarction.

The MRA vessels distal to the occlusion were evaluated as an indication of leptomeningeal collateral supply to the ischemic area. Compared to the contra-lateral normal cerebral hemisphere, a 3-point grading scale was used (0 = none/poor, 1 = fair, 2 = good/normal) (24).

### Statistical analysis

Statistical analysis was performed using the Statistical Product and Service Solutions software (SPSS, Chicago, Ill). Medcalc and GraphPad Prism 8^2^ software packages were used for graph rendering. All clinical and imaging metrics were compared between the good and poor prognosis groups. Student’s *t*-test or Mann–Whitney *U*-test was used for continuous variables with a normal or nonnormal distribution, and the Chi-square test was used for categorical variables. Continuous variables included age, lesion size, rMTT, rTTP, rCBF, rCBV, rMD, and rMK of infarction lesions and peripheral regions. Categorical variables included gender, history of hypertension, diabetes, smoking, drinking, previous stroke, NIHSS score, and grading of collateral circulation. Spearman’s correlations were determined between age, imaging markers, and different prognoses. The chi-square test and Cramer’s V coefficient were determined between gender, all risk factors, NIHSS score, grading of collateral circulation, and different prognoses. The ROC curve was used to compare the predictive efficacy of clinical and imaging metrics on the prognosis of the infarction, and the area under the curve (AUC) was calculated. *p* < 0.05 was considered a significant difference.

## Results

A total of 250 patients with AIS were collected, among which seven cases with surgery or intracranial lesions, 92 cases with small lesions, and 62 cases with bilateral lesions were excluded. Finally, 89 cases (68 males, 21 females; mean age 61.9 ± 9.7 years, age range 37–80 years) were included. A flow chart of the patient inclusion process in the study is depicted in Figure 1. The NIHSS score of all patients was assessed on admission (mean score 4.876 ± 3.867, score range 0–16). Meanwhile, 41 cases were diagnosed as mild stroke by the neurologist, 33 as moderate stroke, 2 as moderate to severe stroke, and the remaining 13 patients had no significant neurological impairment. The collateral circulation status evaluated by MRA was grade 0 in 38 cases, grade 1 in 33 cases, and grade 2 in 18 cases. At 90 days after discharge, 42 patients had a good prognosis and 47 patients had a poor prognosis. The demographic and clinical features of all patients in the two groups are shown in Table 1.

**Figure 2:**
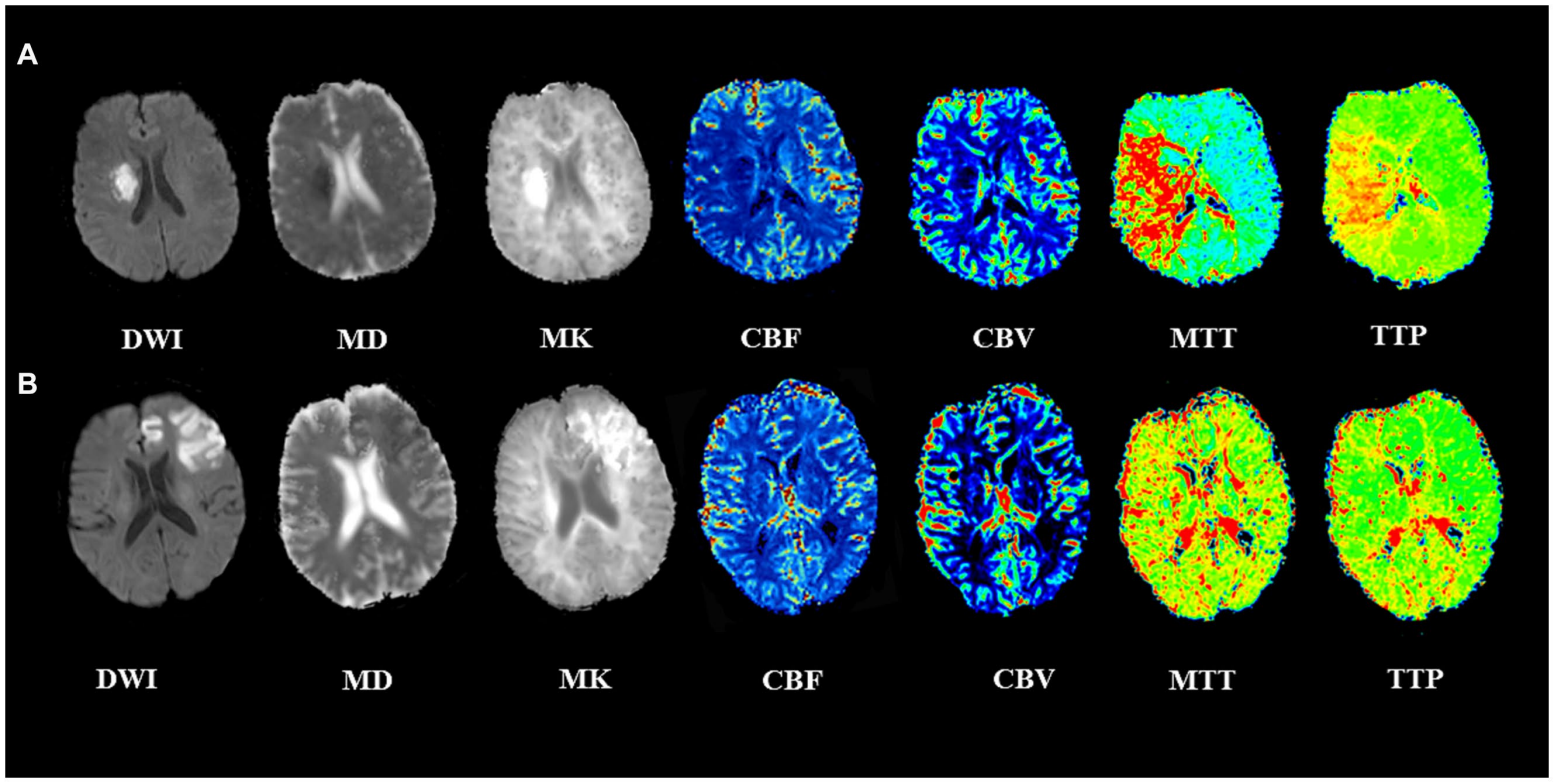
Two representative cases with acute ischemic stroke. **(A)** A 56-year-old man with left limb numbness for 3 days. The lesion adjacent to the right basal ganglia showed a low MD, high MK lesion, with a large area of increased MTT, TTP and decreased CBF. The admission NIHSS score was 3, 90-day mRS score was 4, indicating a poor prognosis. **(B)** A 62-year-old man with dysarthria for 2 h. The lesion at the left frontal cortex showed heterogeneous low MD, high MK, CBF, MTT, and TTP changes were not noticeable. The admission NIHSS score was 10, 90-day mRS score was 1, indicating a good prognosis.

All 89 patients completed imaging examinations successfully. DWI images showed high signal in all cases, and most cases showed low signal lesions on MD images, high signal lesions on MK images, increased MTT, TTP, and decreased CBF. In some cases, MK and MD images showed heterogeneous signals, and CBF changes were not obvious. These patients without significant imaging changes (MK, MD, and CBF) had a better prognosis. Two typical cases are shown in Figure 2.

### Relationship between clinical features and the prognosis

Clinical information included age, gender, risk factors (hypertension, diabetes, previous stroke, smoking, and drinking history), and NIHSS score. As shown in Table 1, age and all history risk factors did not differ significantly with different prognoses. The prognosis of males was worse than that of females (*p* = 0.04). In addition, there was a significant positive correlation between NIHSS score and different prognoses (r = 0.384, *p* < 0.001). Despite having a weak correlation with prognosis, gender cannot be used as an independent indicator of prognosis, among all clinical features.

### Relationship between imaging features of infarction lesions and the prognosis

Infarction was categorized into cortical, subcortical, basal ganglia, and subtentorial based on the locations and blood supply. Other imaging features included lesion size, normalized diffusion (rMD and rMK), perfusion metrics (rMTT, rTTP, rCBF, and rCBV), and collateral circulation status. As shown in Table 1, lesion size and the types of cerebral infarction did not differ significantly with different prognoses. The other imaging metrics were statistically different between the two prognosis groups. rMK (r = 0.545, *p* < 0.001), rMD (r = −0.449, *p* < 0.001), and rCBF (r = −0.348, *p* < 0.001) were significantly correlated with functional prognosis, rMTT (r = 0.261, *p* = 0.01), rTTP (r = 0.325, *p* = 0.001), and rCBV (r = −0.268, *p* = 0.008) were correlated with functional prognosis.

In addition, different collateral circulation status also showed a significant correlation with prognosis (r = 0.477, *p* < 0.001).

### Relationship between imaging features of peripheral regions and the prognosis

As shown in Table 1, The diffusion characteristics (rMD and rMK) of the peripheral regions were not significant to the outcomes (Both *p* > 0.05). The rCBF of peripheral regions was mildly significant to the prognosis (r = −0.278, *p* = 0.011), but the time metrics (rMTT, rTTP) of peripheral regions showed more significant to the final prognosis (r = 0.329, *p* = 0.003 and r = 0.332, *p* = 0.002, respectively). The rCBV of peripheral regions showed no significance to the prognosis (r = −0.125, *p* = 0.263).

### The efficiency of clinical and imaging metrics in predicting the prognosis

**Figure 3:**
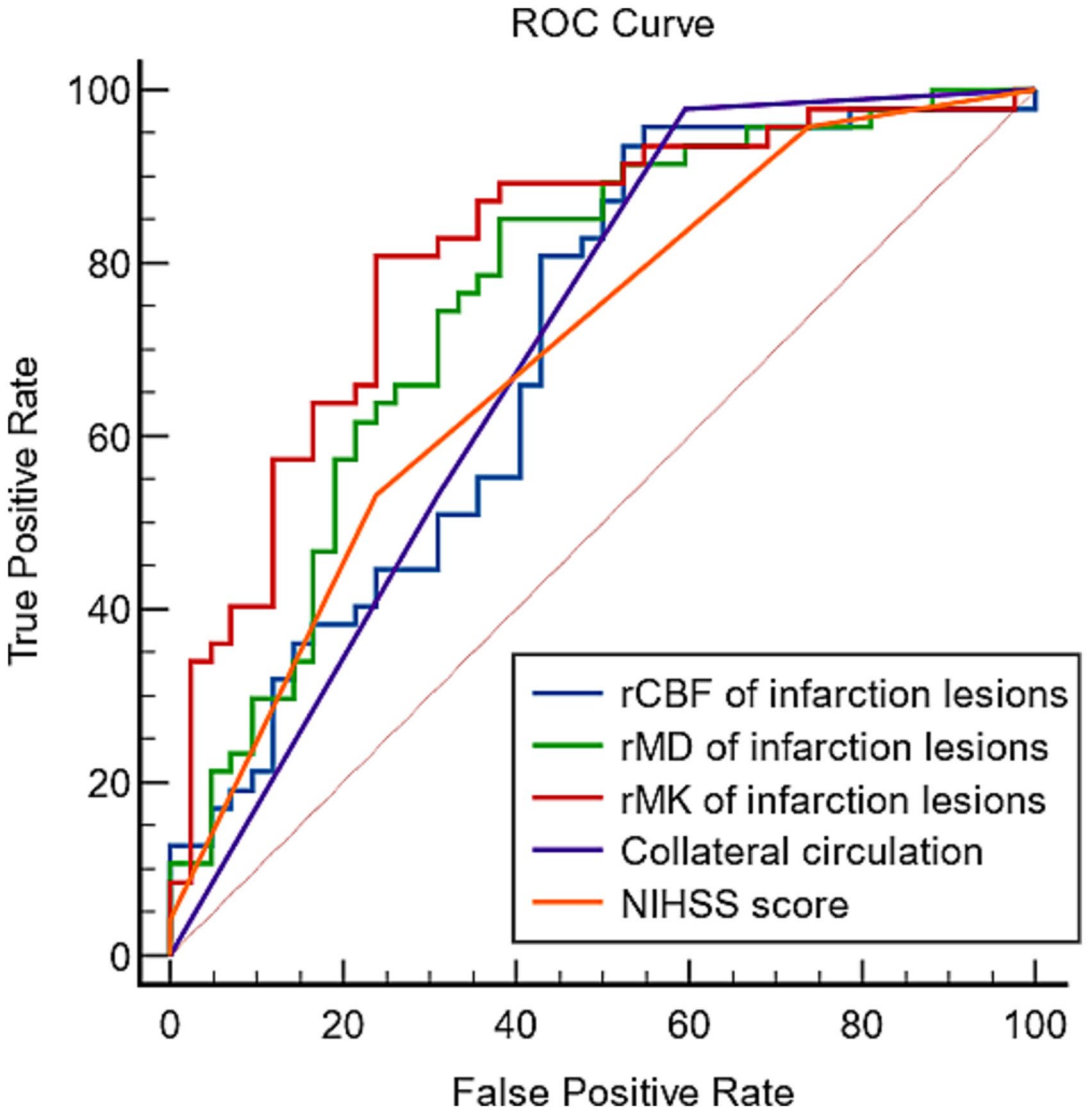
The predictive efficiency of different metrics for the prognosis of strokes.

In all clinical and imaging metrics, the NIHSS score, rMD, rMK, rCBF of infarction lesions, and collateral circulation were most significantly correlated with the prognosis (*p* < 0.001). The ROC curves of these key markers in predicting the prognosis are presented in Figure 3. Areas under the curve of NIHSS score, rMD, rMK, rCBF, and collateral circulation were 0.701, 0.759, 0.815, 0.704, and 0.697, respectively.

## Discussion

AIS is one of the leading causes of disability and a significant cause of mortality among adults (25). The prevalence of AIS is high, and the annual incidence is steadily increasing, bringing a severe burden to patients and society. Therefore, early diagnosis, prognosis assessment, and treatment are essential.

Magnetic resonance imaging (MRI) is a commonly used diagnostic tool for stroke in the clinical setting (15). Imaging indicators are critical and can provide detailed information about the lesions. The prognosis of AIS is highly correlated with the imaging markers. The DWI sequence is widely used in the diagnosis of early cerebral infarction, and it is especially sensitive for detecting the location of cerebral infarction and the size of necrotic lesions in brain tissue (26). In addition to DWI, PWI can non-invasively and intuitively reflect the degree of cerebral blood flow perfusion attenuation. The changes in its perfusion indicators are consistent with the pathophysiological process of cerebral ischemia. PWI can detect a decrease in blood perfusion before DWI detects a cerebral infarction and can be used to assess perfusion improvement before and after cerebral ischemia treatment. Hence, PWI has clinical utility in the early detection and prognosis assessment of cerebral ischemic events (27).

DKI is a kurtosis imaging technology based on DWI and diffusion tensor imaging (DTI), and kurtosis is a statistical metric for quantifying the non-Gaussianity of a probability distribution (10). In normal tissues, the movement of water molecules usually deviates from Gaussian distribution due to the restrictions of the cell membrane and organelle membrane. Different physiological environments have different degrees of deviations from Gaussian distribution, and DKI technology can detect the deviation degree through high b values of diffusion gradients. A more significant deviation leads to a higher MK value. Therefore, the MK value can reflect the restricted degree of movement of water molecules in tissues, and thereby obtain information on subtle structural changes. Previous DKI studies on acute cerebral infarction have found higher MK and lower MD in infarction foci (28, 29). Although various parameters could be obtained from DKI, only MK and MD were mainly used in patients with AIS. Previous studies have shown that AK, RK, and other parameters are insufficient, and MK is the best evaluation parameter for ischemic lesions (30).

Various independent factors influence the prognosis of acute cerebral infarction. Numerous clinical metrics, including the patient’s age, onset time, symptoms, type and size of infarct lesions, concurrent diabetes and other disorders, as well as various treatment options, could affect the prognosis of patients (31, 32). The NIHSS score is frequently employed in the clinical setting to directly reflect patients’ neurological injury status. And the NIHSS score at admission was found to be correlated with the prognosis in this study; while other clinical information was not found to be significant when analyzed as independent variables. Zheng et al. (33) performed multiple evaluations of the NIHSS score after the onset of the disease and found a strong correlation between the NIHSS score at 7 days after the onset and the long-term prognosis of the patients. For imaging metrics, perfusion metrics that reflect the blood flow status of the infarct tissue are correlated with prognosis. In addition, other factors such as cell tolerance to ischemia and the presence or absence of penumbra may also affect the prognosis of patients. Due to the limitation of the examination technique, the mismatch between DWI and PWI cannot obtain the true penumbra, so the correlation between the penumbra condition around the lesion and different prognosis was not performed in this study. For the status of collateral circulation around the lesion, we found a significant correlation between it and prognosis, which is also consistent with some studies. The MK and MD metrics have a higher correlation with the prognosis and are apparent indicators of the degree of cell ischemia. They can indicate the swelling status of the cells in the infarct tissues after ischemia. The MK value has higher sensitivity and specificity than the MD value for predicting a patient’s prognosis. The inability of MD to fully capture structural complexity may be because it is influenced not only by tissue complexity but also by molecule concentration and other factors (9). Other studies also confirmed that MK has more specificity in determining tissue microstructural changes than MD (34).

Different cerebral infarction types and blood supply regions might have different sizes of infarction, as well as distinct imaging appearances and prognoses, which could be related to variations in local structure, blood supply, and peripheral circulation (35). However, our study did not find significant differences in prognosis among different types of cerebral infarctions, which may be due to the dependence of infarction types on other factors, particularly infarct size at admission.

This study had several limitations. First, the sample size was small. Due to the complexity of the clinical factors, the exclusion criteria were strict. For practical reasons, we could only include the lesions with diameter > 1 cm in the study, which might cause a bias in the results. Hence, large sample and multi-center studies are required. Second, the MRI examinations were performed on the second or third day of admission to avoid delays in emergency treatment. The imaging characteristics of the acute stage need to be further studied. Third, this study focused on the clinical history and key imaging indicators to evaluate the prognosis, but other factors, such as the laboratory tests, were not analyzed. Further studies are needed to confirm the results of this preliminary study.

## Conclusion

Perfusion and diffusion metrics could reflect the microstructure and blood flow characteristics of the lesion, which were the key factors for the salvage ability and prognosis of the infarction tissue. The characteristics of the infarction core and peripheral regions have different effects on the outcomes. Diffusion of infarction core has strong relations with the prognosis, whereas the time metrics (MTT, TTP) were more important for peripheral regions. MK had a more significant association with prognosis than MD. The NIHSS score at admission could reflect the functional involvement and was correlated with patients’ outcomes. These factors were the primary clinical and imaging markers influencing the prognosis of cerebral infarction patients.

## Data availability statement

The raw data supporting the conclusions of this article will be made available by the authors, without undue reservation.

## Ethics statement

The studies involving humans were approved by the Ethics Committee of Tianjin First Central Hospital. The studies were conducted in accordance with the local legislation and institutional requirements. The participants provided their written informed consent to participate in this study.

## Author contributions

CP: Conceptualization, Data curation, Investigation, Methodology, Software, Writing – original draft, Writing – review & editing. CH: Conceptualization, Data curation, Investigation, Methodology, Software, Writing – original draft. HL: Investigation, Software, Writing – original draft. XL: Funding acquisition, Writing – review & editing. WH: Conceptualization, Writing – review & editing. JL: Project administration, Supervision, Validation, Writing – review & editing. JY: Conceptualization, Formal Analysis, Funding acquisition, Investigation, Methodology, Project administration, Resources, Supervision, Visualization, Writing – review & editing.

## Footnotes

^1^ https://www.nitrc.org/projects/dke/

^2^ https://www.graphpad.com

## References

[1] Herpich F Rincon F. Management of Acute Ischemic Stroke. Crit Care Med. (2020) 48:1654–63. doi: 10.1097/CCM.0000000000004597, PMID: 32947473 PMC7540624

[2] Sennfält S Pihlsgård M Norrving B Ullberg T Petersson J. Ischemic stroke patients with prestroke dependency: characteristics and long-term prognosis. Acta Neurol Scand. (2021) 143:78–88. doi: 10.1111/ane.13328, PMID: 32738814 PMC7754457

[3] Mozaffarian D Benjamin EJ Go AS Arnett DK Blaha MJ Cushman M. Heart disease and stroke statistics—2016 update: a report from the American Heart Association. Circulation. (2016) 133:e38–e360. doi: 10.1161/CIR.0000000000000350, PMID: 26673558

[4] Ryu W-S Hong K-S Jeong S-W Park JE Kim BJ Kim J-T. Association of ischemic stroke onset time with presenting severity, acute progression, and long-term outcome: a cohort study. PLoS Med. (2022) 19:e1003910. doi: 10.1371/journal.pmed.1003910, PMID: 35120123 PMC8815976

[5] Shuaib A Lees KR Lyden P Grotta J Davalos A Davis SM. NXY-059 for the treatment of acute ischemic stroke. N Engl J Med. (2007) 357:562–71. doi: 10.1056/NEJMoa070240, PMID: 17687131

[6] Ntaios G Lip GYH Vemmos K Koroboki E Manios E Vemmou A. Age- and sex-specific analysis of patients with embolic stroke of undetermined source. Neurology. (2017) 89:532–9. doi: 10.1212/WNL.0000000000004199, PMID: 28687720 PMC5562957

[7] Chemmanam T Campbell BCV Christensen S Nagakane Y Desmond PM Bladin CF. Ischemic diffusion lesion reversal is uncommon and rarely alters perfusion-diffusion mismatch. Neurology. (2010) 75:1040–7. doi: 10.1212/WNL.0b013e3181f39ab6, PMID: 20720188

[8] Schaefer PW Grant PE Gonzalez RG. Diffusion-weighted MR imaging of the brain. Radiology. (2000) 217:331–45. doi: 10.1148/radiology.217.2.r00nv24331 11058626

[9] Jensen JH Helpern JA Ramani A Lu H Kaczynski K. Diffusional kurtosis imaging: the quantification of non-gaussian water diffusion by means of magnetic resonance imaging. Magn Reson Med. (2005) 53:1432–40. doi: 10.1002/mrm.20508, PMID: 15906300

[10] Jensen JH Helpern JA. MRI quantification of non-Gaussian water diffusion by kurtosis analysis. NMR Biomed. (2010) 23:698–710. doi: 10.1002/nbm.1518, PMID: 20632416 PMC2997680

[11] Thijs VN Lansberg MG Beaulieu C Marks MP Moseley ME Albers GW. Is early ischemic lesion volume on diffusion-weighted imaging an independent predictor of stroke outcome?: a multivariable analysis. Stroke. (2000) 31:2597–602. doi: 10.1161/01.STR.31.11.2597 11062281

[12] Guo Y Zhang Z Zhang G Kong L Rao H Chen W. Evaluation of mean diffusion and kurtosis MRI mismatch in subacute ischemic stroke: comparison with NIHSS score. Brain Res. (2016) 1644:231–9. doi: 10.1016/j.brainres.2016.05.020, PMID: 27208488

[13] Zhu L-H Zhang Z-P Wang F-N Cheng Q-H Guo G. Diffusion kurtosis imaging of microstructural changes in brain tissue affected by acute ischemic stroke in different locations. Neural Regen Res. (2019) 14:272–9. doi: 10.4103/1673-5374.244791, PMID: 30531010 PMC6301161

[14] Donnan GA Baron J-C Ma H Davis SM. Penumbral selection of patients for trials of acute stroke therapy. Lancet Neurol. (2009) 8:261–9. doi: 10.1016/S1474-4422(09)70041-9 19233036

[15] Nael K Kubal W. Magnetic resonance imaging of acute stroke. Magn Reson Imaging Clin N Am. (2016) 24:293–304. doi: 10.1016/j.mric.2015.11.002 27150320

[16] Straka M Albers GW Bammer R. Real-time diffusion-perfusion mismatch analysis in acute stroke. J Magn Reson Imaging. (2010) 32:1024–37. doi: 10.1002/jmri.22338, PMID: 21031505 PMC2975404

[17] Zhou Y Zhang S Lou M. Imaging markers in acute phase of stroke: implications for prognosis. Brain Hemorrhages. (2020) 1:19–23. doi: 10.1016/j.hest.2019.12.002

[18] Appelros P Nydevik I Viitanen M. Poor outcome after first-ever stroke: predictors for death, dependency, and recurrent stroke within the first year. Stroke. (2003) 34:122–6. doi: 10.1161/01.STR.0000047852.05842.3C 12511762

[19] Saber H Saver JL. Distributional validity and prognostic power of the National Institutes of Health stroke scale in US administrative claims data. JAMA Neurol. (2020) 77:606–12. doi: 10.1001/jamaneurol.2019.5061, PMID: 32065612 PMC7042858

[20] Huybrechts KF Caro JJ Xenakis JJ Vemmos KN. The prognostic value of the modified Rankin scale score for long-term survival after first-ever stroke. Cerebrovasc Dis. (2008) 26:381–7. doi: 10.1159/000151678, PMID: 18753743

[21] Chen Y Nguyen TN Mofatteh M Abdalkader M Wellington J Yan Z. Association of Early Increase in body temperature with symptomatic intracranial hemorrhage and unfavorable outcome following endovascular therapy in patients with large vessel occlusion stroke. J Integr Neurosci. (2022) 21:156. doi: 10.31083/j.jin2106156, PMID: 36424759

[22] Sun Y Jou E Nguyen TN Mofatteh M Liang Q Abdalkader M. Predictors of futile recanalization after endovascular treatment in acute ischemic stroke: a multi-center study. Front Neurosci. (2023) 17:1279366. doi: 10.3389/fnins.2023.1279366, PMID: 38089974 PMC10713826

[23] Tu W-J Zhao S-J Xu D-J Chen H. Serum 25-hydroxyvitamin D predicts the short-term outcomes of Chinese patients with acute ischaemic stroke. Clin Sci. (2014) 126:339–46. doi: 10.1042/CS20130284, PMID: 24020395

[24] Ernst M Forkert ND Brehmer L Thomalla G Siemonsen S Fiehler J. Prediction of infarction and reperfusion in stroke by flow- and volume-weighted collateral signal in MR angiography. AJNR Am J Neuroradiol. (2015) 36:275–82. doi: 10.3174/ajnr.A4145, PMID: 25500313 PMC7965659

[25] Jadhav AP Desai SM Liebeskind DS Wechsler LR. Neuroimaging of acute stroke. Neurol Clin. (2020) 38:185–99. doi: 10.1016/j.ncl.2019.09.004 31761058

[26] Keir SL Wardlaw JM. Systematic review of diffusion and perfusion imaging in acute ischemic stroke. Stroke. (2000) 31:2723–31. doi: 10.1161/01.STR.31.11.2723 11062301

[27] Quarles CC Bell LC Stokes AM. Imaging vascular and hemodynamic features of the brain using dynamic susceptibility contrast and dynamic contrast enhanced MRI. NeuroImage. (2019) 187:32–55. doi: 10.1016/j.neuroimage.2018.04.069, PMID: 29729392 PMC6538021

[28] Grinberg F. Ciobanu L. Farrher E., &. Shah N. J. (2012). Diffusion kurtosis imaging and log-normal distribution function imaging enhance the visualisation of lesions in animal stroke models. NMR Biomed, 25, 1295–1304. doi: 10.1002/nbm.2802 22461260

[29] Jensen JH Falangola MF Hu C Tabesh A Rapalino O Lo C. Preliminary observations of increased diffusional kurtosis in human brain following recent cerebral infarction. NMR Biomed. (2011) 24:452–7. doi: 10.1002/nbm.1610, PMID: 20960579 PMC3549661

[30] Guo Y-L Li S-J Zhang Z-P Shen Z-W Zhang G-S Yan G. Parameters of diffusional kurtosis imaging for the diagnosis of acute cerebral infarction in different brain regions. Exp Ther Med. (2016) 12:933–8. doi: 10.3892/etm.2016.3390, PMID: 27446298 PMC4950828

[31] Appelros P Stegmayr B Terént A. A review on sex differences in stroke treatment and outcome: sex differences in stroke treatment and outcome. Acta Neurol Scand. (2009) 121:359–69. doi: 10.1111/j.16000404.2009.01258.x 20002005

[32] Leker RR Cohen JE Horev A Tanne D Orion D Raphaeli G. Impact of previous stroke on outcome after thrombectomy in patients with large vessel occlusion. Int J Stroke. (2019) 14:887–92. doi: 10.1177/1747493019841244, PMID: 30947643

[33] Lai Y Jou E Mofatteh M Nguyen TN Ho JSY Diana F. 7-day National Institutes of Health stroke scale as a surrogate marker predicting ischemic stroke patients’ outcome following endovascular therapy. Transl Neurosci. (2023) 14:20220307. doi: 10.1515/tnsci-2022-0307, PMID: 37873059 PMC10590605

[34] Umesh Rudrapatna S Wieloch T Beirup K Ruscher K Mol W Yanev P. Can diffusion kurtosis imaging improve the sensitivity and specificity of detecting microstructural alterations in brain tissue chronically after experimental stroke? Comparisons with diffusion tensor imaging and histology. NeuroImage. (2014) 97:363–73. doi: 10.1016/j.neuroimage.2014.04.013, PMID: 24742916

[35] Chng SM Petersen ET Zimine I Sitoh Y-Y Lim CCT Golay X. Territorial arterial spin labeling in the assessment of collateral circulation: comparison with digital subtraction angiography. Stroke. (2008) 39:3248–54. doi: 10.1161/STROKEAHA.108.520593, PMID: 18845805

